# Hermaphrodite life history and the maintenance of partial selfing in experimental populations of Caenorhabditis elegans

**DOI:** 10.1186/1471-2148-14-117

**Published:** 2014-06-02

**Authors:** Sara Carvalho, Patrick C Phillips, Henrique Teotónio

**Affiliations:** 1 Instituto Gulbenkian de Ciência, Apartado 14, P-2781-901 Oeiras, Portugal; 2 Institute for Ecology and Evolution, 5289 University of Oregon, Eugene, OR 97403, USA; 3 École Normale Supérieure, Institut de Biologie de l’ENS (IBENS), and Inserm U1024, and CNRS UMR 8197, F-75005 Paris, France

## Abstract

**Background:**

Classic population genetics theory predicts that mixed reproductive systems, where self reproduction (selfing) and outcrossing co-exist, should not be as common as they are in nature. One means of reconciling theory with observations is to recognize that sexual conflict between males and hermaphrodites and/or constraints in the allocation of resources towards sex functions in hermaphrodites can balance the fitness components of selfing and outcrossing.

**Results:**

Using experimental evolution in *Caenorhabditis elegans*, we test whether the adaptive maintenance of partial selfing is due to sexual conflict and/or to the evolution of sex allocation towards male function in hermaphrodites. For this, we characterized the reproductive schedule and longevity patterns in hermaphrodites under selfing and under outcrossing with naïve males that did not have the opportunity to evolve with them. A shift in reproductive schedule towards earlier reproduction would be indicative of adaptation in our imposed life-cycle, while longevity is expected to evolve as a response to the harm that males impinge on hermaphrodites upon mating. To determine adaptation in the absence of constraints in sex allocation, we also characterized the life history of females that reproduced during experimental evolution through obligate mating with males. As expected with adaptation, we find that after 100 generations of experimental evolution, selfing hermaphrodites and females showed improved reproduction at earlier ages. We did not observe similar reproductive shifts in outcrossed hermaphrodites. We further find increased longevity in outcrossed females after evolution but not in outcrossed hermaphrodites, a result that indicates that sexual conflicts were likely more prevalent under male–female evolution than under male-hermaphrodite evolution.

**Conclusions:**

Taken together, our findings suggest that the adaptive maintenance of partial selfing during *C. elegans* experimental evolution resulted from the evolution of sex allocation towards male function in hermaphrodites.

## Background

Classic population genetic models for the evolution of different reproductive modes within populations usually predict bi-modal distributions of predominant outcrossing or selfing [1-3]; but see, e.g., [4-8]. Selection against deleterious recessive alleles is more effective in populations that reproduce more frequently via selfing than outcrossing because selfing increases segregation of homozygotes, which leads to the evolution of higher selfing rates. In contrast, populations that reproduce predominantly via outcrossing can maintain higher loads of recessive deleterious alleles, which should make those populations resistant to invasion by selfers and keep selfing rates low.

In contrast to theoretical expectations, many natural populations of plants and animals exhibit mixed reproductive systems with partial selfing [9,10]. One means of reconciling empirical observations with theory is to recognize that the fitness components expressed under selfing or outcrossing might tradeoff with one another, e.g., [11-13], and thereby add an additional layer of complexity to the population genetic dynamics. In plants, for example, the balance between reproduction via selfing and pollen export for outcrossing can be influenced by flower morphology, relative flower position within the individual, and pollinator availability [11,14-18].

There are two causes for tradeoffs between selfing and outcrossing fitness components. First, tradeoffs can be explained by the existence of sexual conflicts that lead to the antagonistic coevolution between individuals of different sexes. For example, in male-hermaphrodite reproduction systems, increased male mating success may be associated with hermaphrodites becoming more resistant to mating if increased male performance is somehow harmful to the hermaphrodite. The hypothesis of a sexual conflict therefore predicts negative genetic correlations between the fitness components of individuals of different sexes. Sexual conflict has largely been described for obligate outcrossers, e.g., [19-21], but could also be important for partial selfers [22-24].

Second, tradeoffs between fitness components derived from reproduction via selfing and outcrossing may be explained by constraints in the allocation of developmental, physiological or ecological resources between male and female functions, particularly in hermaphrodites [18,25-28]. This would be the case, for example, if increased proliferation of ovules or oocytes were associated with decreased proliferation of pollen or self-sperm during gametogenesis, and vice-versa. The hypothesis of a sex allocation therefore predicts negative genetic correlations between the sex-specific fitness components in hermaphrodites.

The two hypothesis explaining tradeoffs between selfing and outcrossing fitness components are not mutually exclusive and for this reason empirically determining whether the evolution of a sexual conflict or the evolution of sex allocation underlie the maintenance of partial selfing has been difficult. In this regard, the androdioecious (male-hermaphrodite) nematode *Caenorhabditis elegans* may shed light, since over the last decade it has become an experimental model for the evolution of outcrossing and selfing [12,29-32]. Interestingly, although the analysis of molecular variation suggests that outcrossing via males is rare in nature [33-35], outcrossing rates exceeding those expected under mutation-genetic drift equilibrium are frequently observed when populations are maintained in the laboratory under novel conditions [36-38].

The androdioecious reproductive system of *C. elegans* is characterized by the existence of hermaphrodites that are able to cross-fertilize their oocytes only when mated with males [39]. It is therefore plausible that partial selfing is maintained within experimental populations via sexual conflict. In fact, mating with males is known to have harmful effects on the longevity of hermaphrodites [40], and, at the species-wide level, hermaphrodites have lost the ancestral female ability to attract males and respond to male mating behaviours [41,42]. In the presence of sexual conflict, the evolution of increased male mating success would thus be expected to be countered by evolutionary responses in hermaphrodite longevity. The experimental populations used here, which maintain partial selfing, have been shown to evolve increased male reproductive success [38], but it is unknown whether or not hermaphrodites responded by increased resistance to mating with males, cf., [32,38], a result that would be consistent with the sexual conflict hypothesis.

*C. elegans* is also characterized by protandrous selfing, since hermaphrodites first produce sperm and then irreversibly switch to oogenesis before maturity [43]. Depending on the frequency of males and time of reproduction, selfing will generally precede outcrossing [12], though once hermaphrodites are mated male sperm outcompete self-sperm [44]. Since hermaphrodites cannot mate with each other, it is possible that the common developmental program for sperm and oocytes in hermaphrodites results in negative genetic correlations between selfing and outcrossing fitness components [45,46]. Thus, in addition to possible sexual conflict, the maintenance of partial selfing during experimental evolution may also be due to an evolutionary shift in sex allocation towards male function in hermaphrodites.

Here, we aim to determine whether the evolution of a sexual conflict or the evolution of sex allocation can explain the maintenance of the 50% outcrossing rate observed during the 100 generations of experimental evolution in male-hermaphrodite androdioecious populations with standing genetic diversity, as reported in [38]. We compare evolutionary changes in the reproduction and longevity patterns of obligate-selfing hermaphrodites with those of obligate-outcrossing hermaphrodites mated with naïve males with which they have not evolved. The use of naïve (tester) males allows the evolutionary response of hermaphroditic function to be tested directly. As a control, we also measured the reproduction and longevity patterns of females from male–female (dioecious) populations evolved under the same environmental conditions of the male-hermaphrodite (androdioecious) populations using the same tester males as those employed in assaying outcrossed hermaphrodites. We thus compare the reproduction and longevity patterns of individuals subject to either selfing or outcrossing: selfed hermaphrodites, outcrossed hermaphrodites and outcrossed females. Evolutionary response was evaluated by contemporaneously characterizing reproduction and longevity in generation 0 and generation 100 individuals.

According to life-history theory, adaptation under our experimental evolution conditions of 4-day discrete and non-overlapping generations is expected to shift reproduction schedules towards earlier ages [47,48]. This is because, under selfing or outcrossing, the majority of reproduction in *C. elegans* hermaphrodites/females occurs after day 4, so a shift towards early life reproduction should be favoured by selection. We use the fact that the evolutionary changes observed for females provide the null expectation for the effects of an on-going sexual conflict in the absence of sex allocation constraints to test the following hypotheses.

First, if a sexual conflict is important for structuring the evolutionary response, then derived generation 100 females should evolve to be become better at resisting the effects of male mating than ancestral females, and so female longevity (a proxy for the cumulative effects of negative interactions between the sexes) should also increase with experimental evolution. If a sexual conflict explains partial selfing in the male-hermaphrodite populations, then evolved hermaphrodites and females tested under similar conditions should both show increased resistance to mating (i.e., increased longevity).

Second, we expect generation 100 females to display increased embryo production and/or survivorship early in life than ancestral females. We also expect such an evolutionary response in hermaphrodites when they self and outcross. However, if evolution of sex allocation towards male function explains partial selfing in the male-hermaphrodite populations, the investment made early in life relative to lifetime reproduction should increase only when hermaphrodites self. For outcrossed hermaphrodites, increased allocation of resources towards male function is expected to be neutral or disadvantageous and thus the relative reproductive investment early in life should not respond during experimental evolution.

## Results

### Reproductive schedules

The individual reproductive schedule measurements are summarized in Additional file 1: Figure S1 and Additional file 1: Table S1 (full dataset before quality control can be found in [49]). Breeding mode influences the lifetime production of embryo and adult progeny. Selfing hermaphrodites start reproducing between day 3 and day 4 of the life-cycle and by day 7 have mostly ceased to reproduce. Outcrossed hermaphrodites and females on the other hand start reproducing between day 3 and 4, but only cease reproduction by day 9. As a consequence, outcrossed individuals have higher total lifetime reproductive output. These reproduction patterns are explained by the fact that *C. elegans* hermaphrodites are self sperm limited. Also as expected, mating with males early in life greatly decreases the average longevity of hermaphrodites and females, when compared with hermaphrodites undergo selfing.

### Evolution of fecundity

**Figure 1:**
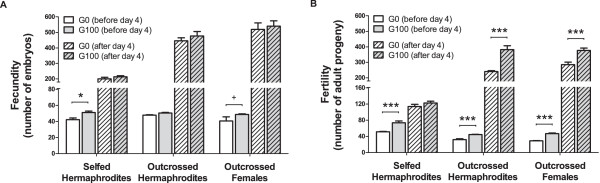
**Evolution of fecundity and fertility.** Bars represent the average individual fecundity (number of embryo progeny; panel **A)** or fertility (number of adult progeny; **B)** before or after day 4 of the life-cycle, the day passaging occurred during experimental evolution. Empty bars show results for samples of the ancestral populations (G0) and grey bars for derived populations after 100 generations of experimental evolution (G100). Individuals were enforced either to self or to outcross with tester males. Hermaphrodites came from male-hermaphrodite populations, which had maintained stable 50% of outcrossing during experimental evolution. Females came from male–female populations. Error bars denote one standard mean error among the three blocks done. Significant evolutionary responses (differences between G100 and G0) are indicated with: +, p < 0.1; *, p ≤ 0.05, ** p ≤ 0.01; ***; p ≤ 0.001. Y-axes are truncated for clarity.

Selfed hermaphrodites from male-hermaphrodite populations at G100 lay significantly more embryos before day 4 than their ancestors (Figure 1A, t_142.8_ = 2.4, p = 0.02; see Methods for a description of statistical modelling). After day 4, however, we measured no evolutionary changes in fecundity. Outcrossed hermaphrodites showed no differences whether they came from G0 or G100 populations, at either period of the life-cycle. Outcrossed females from male–female populations at (G100) may lay a significant higher number of embryos before day 4 than their ancestrals (t_149.7_ = 1.8, p = 0.08), but not after day 4.

### Evolution of fertility

For early life fertility, before day 4, we observed significant evolutionary increases in all treatments (Figure 1B): for selfed hermaphrodites t_112.3_ = 7.9, p < 0.001, for outcrossed hermaphrodites t_142.55_ = 4.3, p < 0.001, for outcrossed females t126.9 = 6.4, p < 0.001. For late fertility, after day 5, selfed hermaphrodites did not show any changes relative to their ancestors. On the other hand, outcrossed hermaphrodites evolved to be more fertile than their ancestors (t_126.2_ = 9.9, p < 0.001), and so did outcrossed females (t_123.6_ = 5.1, p < 0.001). In this later treatment, we found that the linear mixed model’s (LMM) residuals did not follow normality (Shapiro-Wilk W = 0.97, p = 0.012), though we confirmed the significance of evolutionary responses with generalized linear mixed models (GLMM) employing Poisson errors.

### Evolution of relative reproductive investment

**Figure 2:**
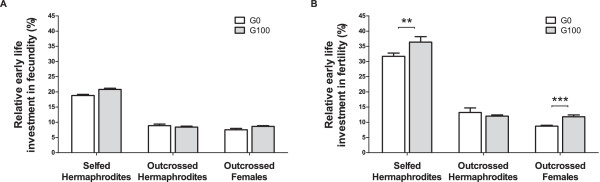
**Evolution of early life relative reproductive investment.** Bars represent the average individual relative reproductive investment in fecundity **(A)** or fertility **(B)** until day 4, calculated as the number of embryo or adult progeny over the total lifetime number of embryo or adult progeny, respectively, from Figure 1. The three treatment groups are shown: selfed and outcrossed hermaphrodites, outcrossed females. Error bars denote one standard mean error among the three blocks done. Significant evolutionary responses are indicated with: **p ≤ 0.01; ***; p ≤ 0.001.

In order to compare the evolutionary responses among individuals reproducing under selfing or outcrossing, we calculated the proportion of embryos and adults that were measured before day 4 over total lifetime numbers, which we defined as relative reproductive investment early in life. For early life investment in fecundity, we were unable to measure significant evolutionary changes (Figure 2A). In contrast, there was a significant evolutionary increase in early life fertility investment in hermaphrodites that selfed (Figure 2B; t_136_ = 2.6, p = 0.01). Outcrossed hermaphrodites did not show any evolutionary change early life fertility investment. Outcrossed females, like selfed hermaphrodites, showed an increase in early life fertility investment (t_136_ = 4, p = 0.001). Selfed hermaphrodites and outcrossed females therefore showed a shift towards earlier relative reproductive success, when compared to their ancestors.

### Evolution of longevity

**Figure 3:**
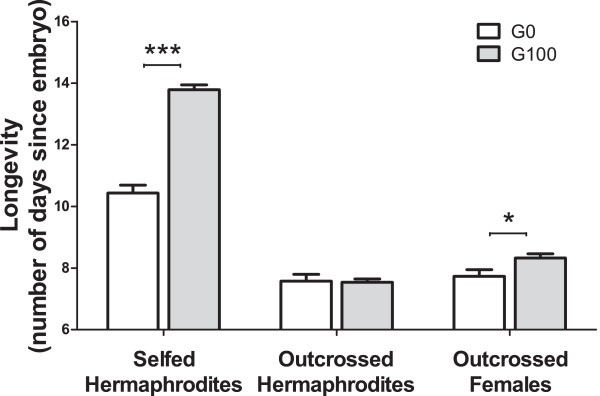
**Evolution of longevity.** Bars represent the average individual longevity measuring in the reproductive schedule assay. The three treatment groups are shown: selfed and outcrossed hermaphrodites, outcrossed females. Error bars denote one standard mean error among the three blocks done. Significant evolutionary responses are indicated with: *, p ≤ 0.05, **p ≤ 0.01; ***; p ≤ 0.001.

Individuals measured for fecundity and fertility in the reproductive schedule assay were also scored for longevity. Selfed hermaphrodites from G100 populations had a significantly higher longevity than their ancestrals (Figure 3, t_102.3_ = 4.5, p < 0.001) and although the LMM residuals did not follow normality (W = 0.95, p = 0.001), we confirmed the significance of this longevity increase with evolution when employing GLMM with binomial errors. Outcrossed hermaphrodites from G100 population did not show any longevity differences from their ancestors. In contrast, outcrossed females from G100 populations showed higher longevities than their ancestors (t_83.1_ = 2.2, p = 0.03)

### Evolution of longevity in the absence of mating

**Figure 4:**
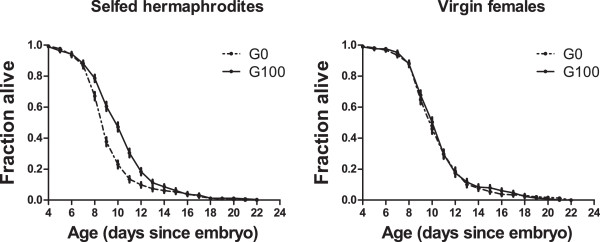
**Longevity in the absence of mating.** Age-specific Kaplan-Meyer survival rates of selfed hermaphrodites (left) and virgin females (right) of ancestral G0 populations (dashed lines) and derived G100 populations (full lines). Error bars denote one standard deviation of the estimated survival function.

To test that the evolutionary increase in female longevity was due to an evolutionary increase in their resistance to mating with males, we conducted longevity assays as before but in the absence of mating. Analysis of this assay indicated that the survival rates of unmated (virgin) females did not show any evolutionary response (Figure 4). Confirming the findings from the reproductive schedule assay, we continued to find that the risk of dying with increased age was reduced in evolved unmated (selfed) hermaphrodites (mortality rates H = 0.56 and H = 0.589, both p < 0.001, for replicates A2 and A3, respectively; not significant for the A1 replicate).

## Discussion

We have previously shown that male-hermaphrodite populations of *C. elegans*, characterized by having standing genetic diversity and cultured at high population sizes in a 4-day non-overlapping life-cycle, maintained partial selfing at roughly 50% during the 100 generations of experimental evolution [38]. Such intermediate selfing rates are not expected under classic population genetic models, which posit that the evolution of inbreeding depression generated by deleterious recessive alleles is the cause for the evolution of different reproduction modes [1,3,9]. A tradeoff between fitness components under selfing and outcrossing is one way of resolving this conundrum [13]. What might the nature of these tradeoffs be?

Fitness tradeoffs between selfing and outcrossing can result both via antagonistic coevolution between hermaphrodites and males [22,24], and via constraints on sex allocation between male and female function in hermaphrodites [18,25,26,28]. In particular, the existence of sexual conflict predicts negative genetic correlations between the fitness components of different sexes, whereas the existence sex allocation predicts the existence of negative genetic correlations between the fitness components of only one sex, the hermaphrodite. Evolution of a sexual conflict and evolution of sex allocation are not mutually exclusive and therefore can both potentially explain the maintenance of partial selfing.

Starting first with sexual conflict, we found that outcrossing in *C. elegans* reduces the longevity of hermaphrodites/females, as in previous studies, cf., [40,50]. If this cost of mating is maintained in part by antagonistic sexual coevolution, then restricting selection to fitness components expressed early in life (before day 4) should alter the expected equilibrium between early reproduction and the deleterious impact of mating on longevity [19,51]. Consistent with these predictions, evolved females from the male–female populations showed a shift in their relative reproductive investment towards earlier ages and an increase in longevity (see also [48]). This increase in longevity was conditional on the presence of males, as ancestral and derived populations had virgin females with similar longevities. In contrast, hermaphrodites from the male-hermaphrodite experimental evolution populations showed a shift toward earlier selfing but displayed no apparent reduction in the cost of mating with males.

Lack of evolutionary response in the longevity of mated hermaphrodites is not due to a reduced mating performance of the males in their populations, since male reproductive success similarly increased during the experimental evolution of androdioecious and dioecious populations despite sex ratio differences [38]. The responses in females are therefore consistent with the existence of an underlying sexual conflict and antagonistic coevolution with males. Conversely, the lack of longevity responses in mated hermaphrodites suggests that an on-going sexual conflict was not very important for the maintenance of partial selfing.

We also found that the longevity of selfed hermaphrodites greatly increased with experimental evolution. By definition, this result is not due to a reduction in the cost of mating with males and thus it is not related to sexual conflict. Because of inbreeding depression observed in the ancestral population, increased longevity of evolved selfed hermaphrodites is instead probably due to the purging of deleterious alleles under natural selection during experimental evolution [8,52]. A reduction in the cost of reproduction and/or an increase in somatic viability could underlie this reduction in inbreeding depression [53,54]. Disentangling these alternatives would require further investigation by, for example, genetically eliminating gametogenesis. If a reduction in the cost of reproduction explains the evolution of longevity in hermaphrodites when they self, then eliminating gametogenesis would also eliminate the observed longevity differences between ancestral and evolved populations.

Turning to the possibility that partial selfing was maintained because of constraints on sex allocation, general models for the evolution of sex allocation in hermaphrodites indicate that the degree of selfing is inversely correlated to the allocation of reproductive resources to male function [25,26]. Similarly, other models, particularly those developed to explain the evolution of plant reproductive systems (which generally do not ignore the possibility for outcrossing among hermaphrodites), predict stable intermediate selfing rates when there is a positive genetic correlation between the numbers of selfed and outcrossed ovules [9,13,18].

For the male-hermaphrodite populations examined here, it appears that there was evolution of sex allocation through increased allocation of reproductive resources towards male function. We found increased total lifetime progeny production regardless of treatment, which may indicate that there was evolution of increased proliferation of oocytes (instead of increased offspring viability). However, we did not find evidence for an increase in the relative allocation of resources towards oogenesis since the relative reproductive investment made in embryos or adults early in life did not change in outcrossed hermaphrodites.

It could be argued that no early life relative reproductive investment responses in outcrossed hermaphrodites is explained by lower offspring viability, when compared to the offspring viability of selfed hermaphrodites. Particularly, there could have been outbreeding depression because hermaphrodites were mated with unrelated GFP males. Consistent with this idea, wild *C. elegans* isolates show outbreeding depression when crossed with each other [55,56]. We have previously shown, however, that after 100 generations of experimental evolution outbreeding depression is expected to have been mostly resolved by selection [52]. In male–female populations, in contrast, residual outbreeding depression could have been maintained to larger extent than in male-hermaphrodite populations, as selfing facilitated the purging of deleterious recessive alleles [8,52]. Here, females from the male–female populations increased their relative reproductive investment early in life when outcrossed with similar tester males. Together, these findings suggest that the lack of a response in the early life relative reproductive investment of outcrossed hermaphrodites was not due to outbreeding depression.

The relative reproductive investment under conditions of selfing changed in a direction that was adaptive in our 4-day life cycle protocol: a shift towards early life. As oogenesis is a common developmental program to both selfed and outcrossed hermaphrodites, and because a similar response towards early reproduction was not observed in outcrossed hermaphrodites, we can infer that the allocation towards female function did not change during experimental evolution. If the allocation towards female function did not change, the only way to have evolved increased earlier self-reproduction was by the evolution of resource allocation towards male function. Because we did not observe significant increases in the number of embryos during early reproduction, but did see an increase in the number of adult progeny, it seems likely that part of this evolutionary response is in sperm viability. It is also likely that there was evolution of increased rates of self-sperm proliferation and/or the evolution of retarded switching between spermatogenesis and oogenesis (see [48]).

## Conclusions

Mixed mating systems are relatively common in natural populations of animals and plants. Both sexual conflict and the existence of sex-specific resource allocation constraints in hermaphrodites can mediate the fitness tradeoff between selfing and outcrossing, which in turn can explain the adaptive stability of partial selfing. In our *C. elegans* populations, despite the fact that males greatly increased their reproductive success during evolution, the existence of a sexual conflict appears to have contributed little to adaptation and to the maintenance of partial selfing. Instead, the maintenance of partial selfing was likely due to the evolution of increased allocation towards male function in hermaphrodites.

## Methods

### Experimental evolution

The construction of the populations and the experimental evolution design has been detailed [38]. Briefly, an ancestral hermaphroditic population (EEVA0, A0 for short) was generated by the successive pairwise inter-crossing of 16 inbred wild isolates, while an ancestral male–female population (D0) was derived by the recurrent introgression of the *fog-2(q71)* allele into the A0 population for 22 generations. This allele abolishes hermaphrodite spermatogenesis, without apparent consequences for oogenesis, thus transforming wild type hermaphrodites into functional females [57]. Sperm production in males is unaffected. The *fog-2(q71)* allele has been further shown, in our populations, to be of little consequence for male reproductive success or sex ratio distortion [38].

We report the results for three replicates of each reproductive system (A1-3 and D1-3). Replicate populations were cultured for 100 generations at 20°C and 80%RH, using a discrete 4 day non-overlapping life-cycle, with first larval staged (L1) census size of 10^4^ individuals (which is an order of magnitude higher than the effective population sizes of Ne = 10^3^; [8]). At day 1 of our life-cycle, 10^3^ L1-staged worms were seeded in each of ten Petri NGM-lite plates (US Biological) covered with an *E. coli* HT114 lawn that serves as *ad libitum* food. After growth for 3 days, adults from all plates are mixed and killed using a hypochlorite solution and embryos harvested in a M9 solution without food. After 24 h, hatched embryos have become starvation-arrested L1s, which upon appropriate density estimation are seeded into fresh Petri plates with food to constitute the following generation. This life-cycle was repeated 100 times. Under this scheme, interactions between males and hermaphrodites/females should occur between day 3, when maturity in an average individual is reached (C. Braendle, personal communication), and day 4 of the life-cycle, the day of passaging the population to the following generation.

Inbreeding depression is prevalent in ancestral populations due to the disruption of coevolved sets of loci in the wild isolates and exposure of deleterious recessive alleles [52]. During experimental evolution inbreeding depression is nonetheless diminished, particularly in the androdioecious populations [52]. Population genetic data indicate that rates of genetic diversity changes are slower in the later periods of experimental evolution when compared to initial periods, a result suggesting that most adaptation has occurred by generation 100 [8]. Since *C. elegans* hermaphrodites cannot mate with each other and sex segregation ratios are even when there is outcrossing [38], selfing rates in the male-hermaphrodite (androdioecious) populations can be calculated as one minus twice the proportion of males observed [12], assuming no mixed selfing and outcrossed hermaphrodite broods. During A1-3 experimental evolution, selfing rates evolved by natural selection and were stably kept at 50% [38]. As also previously shown, during experimental evolution the relative male reproductive success increased, similarly among androdioecious and dioecious populations [38].

Naïve tester males employed in the assays here presented were derived by the introgression of the transgenic array *ccls4251 (myo3::GFP) *[58] into the A0 population (A0GFP), as described in [38]. This transgenic array is integrated into chromosome I [58]. Individuals from A0GFP express the fully penetrant green fluorescent protein (GFP) in all larval and adult muscle cells.

### Reproductive schedule assays

Frozen stocks of ancestral (G0) and generation 100 (G100) populations were revived (each with >10^3^ individuals) and cultured for two generations under common conditions prior to the life-history assays in order to avoid confounding maternal and grand-maternal environmental effects. The reproductive schedule assays were divided into three blocks, each incorporating the two ancestral populations (A0, D0) and one of the G100 replicates from each reproductive system. In the third generation after revival, at the defined day 3 of the life cycle, 30 L4 larvae to young adult individuals from each population were individually sampled. Each hermaphrodite of the male-hermaphrodite populations was placed in plates alone or with two tester A0GFP males at the same developmental stage. Females from male–female populations were likewise individually placed with two tester males. Assay plates consisted of 6 cm Petri dishes with NGM-lite and a spot of 5 μl of O/N cultures of *E. coli* HT114. We followed a randomized design for setup.

There were three treatments in the reproductive schedule assay: selfed hermaphrodites, outcrossed hermaphrodites and outcrossed females. Regardless of treatment, all individuals were randomly transferred to new assay plates every day until death. Tester males from the outcrossed treatments were removed 2 days after setup. Hermaphrodite/female death was scored when they ceased pharyngeal pumping, when they failed to move upon prodding of the vulva, or when they were not observed for three consecutive days (and hence assumed to have escaped the assay plate). In this latter case, day of death was recorded as the age the individual was last seen alive. At each day, the number of embryos laid on the assay plates was scored under a stereoscope at 40× magnification to provide a measure for fecundity. Further, embryos were allowed to hatch and grow for 3 days and the live adults scored for number and sex. In the case of the outcrossed treatments, broods were also scored for GFP expression, under a under a stereoscope at 60× magnification equipped with an FITC filter.

### Reproductive schedule quality control

As an initial screen, we eliminated individual observations where the count of adult offspring was higher than the count of embryos, as that would imply progeny viabilities higher than 100% (eliminated n = 43). To properly estimate progeny sex ratios, a high number of individuals needed to be counted, so as a second step, we eliminated individuals with less than 30 viable adult progeny (n = 31). We further eliminated all infertile and low fertility individuals. In the case of females, this step allowed us to prevent the inclusion of observations where there was poor tester male sperm insemination. Specifically for the outcrossed hermaphrodites, we next eliminated data where fewer than 10% male offspring were observed in order to match the minimum sex ratio observed in the outcrossed females (n = 4). Outcrossed treatments were checked for GFP-positive broods indicative of successful cross-fertilization, and therefore hermaphrodites with mixed (non-GFP containing) broods were also eliminated. Finally, we eliminated observations from selfed hermaphrodites with more than 1% in their male progeny. This was done because males can only arise in the progeny of selfed hermaphrodites from the rare (10^**-**3^-10^**-**4^) non-disjunction of the X-chromosome during gametogenesis [38], and thus we prevent the inclusion of hermaphrodites that may have been outcrossed to males from their own population, prior to the assay setup.

The sample size, mean and standard deviation of the quality controlled data are presented in Additional file 1: Table S1 and plotted in Additional file 1: Figure S1.

### Analysis of reproductive schedules

For each treatment (selfed hermaphrodites, outcrossed hermaphrodites, outcrossed females), we analysed the number of embryos and number of adult progeny until the day of usual population passage during experimental evolution (day 4 of the life-cycle), the number of embryos and adult progeny after day 4, and longevity. Under our experimental conditions, new generations are established on day 4, and so adaptation is expected to occur through changes in fitness components before day 4, since there is effectively no selection on reproductive output following day 4 [31,59]. The number of embryos is a measure of fecundity and the number of adult progeny is a measure of fertility (as it compounds individual fecundity and respective progeny survivorship until adulthood).

Data were first Anscombe-transformed [x’ = √(x + 3/8)] and linear mixed effect models (LMM) performed (with fixed generation and random block as independent variables) using restricted maximum likelihood methods, as implemented in the *lme4* package of R [60]. Least-square estimates and significance of effects were obtained against t-distributions using the *lsmeans* package of R [61]. Degrees of freedom for these t-tests were asymptotically determined, c.f. [61]. When significant generation effects were inferred at α ≤ 0.05, the residuals of the models were tested for normality with Shapiro-Wilk tests and for homocedasticity with Bartlett’s tests [62]. In case either of these two LMM assumptions were not followed we defined outlier observations as those whose residuals fell outside the 95% confidence limits of the normal cumulative distribution function, as inspected with QQ plots in the *car* package of R [63]. LMMs were then repeated without the outlier observations. If after outlier removal residual distributions still violated either of the two LMM assumptions, we applied generalized linear mixed effect models (GLMM) with Poisson or binomial error distributions to the data, after quality control and without data transformation, to confirm the significance of generation effects obtained with LMM [60]. Log-link or logit-link functions were employed in *lme4* for error modelling in GLMM [60].

The number of embryos or adult progeny at each period is not immediately comparable between selfed and outcrossed hermaphrodites or females because *C. elegans* is self-sperm limited, and because the introgression of the *fog-2(q71)* allele in the male–female ancestral population may have affected the timing of reproduction in our 4-day life-cycle. For these reasons, we also defined relative reproductive investment in fecundity or fertility until day 4 as the proportion of embryos or adult progeny, respectively, over total lifetime number of embryos or adult progeny. This allows us to equate relative reproductive investment with relative reproductive success during experimental evolution and thus with adaptation. Relative reproductive investment data was angular-transformed (x’ = asin√x) before modelling with LMM and GLMM.

For the figures, we plot the mean values among the measurements made in the three blocks after quality control and LMM analysis, with errors indicating one standard error of the mean among blocks.

### Longevity assay in the absence of mating

To determine whether the observed longevity changes were due to a response to the cost of mating with males, and not a response in the cost of reproduction [53,54], we determined the longevity of hermaphrodites and females in the absence of mating. This longevity assay was carried out in two time blocks, each including the ancestral (A0, D0) and the three derived populations from each reproduction system (A1-3, D1-3). Following a similar procedure to that of the reproductive schedule assays, in the third generation after reviving the frozen population samples, day 3 hermaphrodites or females were individually placed in each well, containing 3.5 mL of NGM-lite with 5 μl of *E. coli* HT114, of 12-well cell culture plates. To prevent density effects caused by progeny growth, individuals were transferred daily to new wells until cessation of hermaphrodite selfing; following this period they were transferred every two days. Females were transferred in a similar schedule. Age of death was scored as in the reproductive schedule assays.

### Analysis of longevity in the absence of mating

Females that had progeny were removed, as were hermaphrodites with male progeny. Sample sizes were of 329 individuals for the A0 population, and 99, 107, and 107 for each of the A1-3 populations; 284 for the D0 population, 88, 86, and 88 for each of the D1-3 populations.

Age-specific survival rates were calculated following the Kaplan-Meyer estimator, and mortality rates as the hazard ratio (H) using the Nelson-Aalen estimator [64]. Right-censoring of the data was employed for both estimates. The Cox proportional hazards regression model was used to test for differences in mortality rates between G100 populations and G0 ancestors (separately for hermaphrodites and females). In each model we estimated the effects of generation with a separate hazard ratio being fitted to each replicate population by defining them as strata. The Efron method was used to handle ties. All calculations were performed using the *survival* package in R [65].

## Availability of Supporting Data

The data sets supporting the results of this article are available from the Dryad Digital Repository: doi:10.5061/dryad.bq280.

## Competing interests

We have no competing interests to declare.

## Authors’ contributions

SC conducted the life-history assays. SC and HT performed data analysis. SC, PCP and HT designed the project and wrote the manuscript. All authors read and approved the final manuscript.

## Supplementary Material

Additional file 1 Reproductive schedule assay sample sizes and summary statistics. Click here for file
