# Supplementary material for: Hermaphrodite life history and the maintenance of partial selfing in experimental populations of Caenorhabditis elegans
Source: BMC Evol Biol. 2014 Jun 2;14:117. doi: 10.1186/1471-2148-14-117 (PMC4052797; doi:10.1186/1471-2148-14-117)
Supplement: Additional file 1 — Reproductive schedule assay sample sizes and summary statistics. [file 1471-2148-14-117-S1.pdf]

Additional Materials

Hermaphrodite life history and the maintenance of partial selfing in experimental populations of Caenorhabditis elegans

Sara Carvalho[1], Patrick C. Phillips[2], Henrique Teotónio[1,3§]

[1]Instituto Gulbenkian de Ciência, Apartado 14, P-2781-901 Oeiras, Portugal.

[2]Institute for Ecology and Evolution, 5289 University of Oregon Eugene, OR 97403, U.S.A.

[3] École Normale Supérieure, Institut de Biologie de l´ENS (IBENS), and Inserm U1024, and CNRS UMR 8197, F-75005 Paris, France

[§]Corresponding author

Contents:

This file contains Supplementary Figure 1 and Supplementary Table 1, with the sample sizes and summary statistics of the reproductive schedule assay.

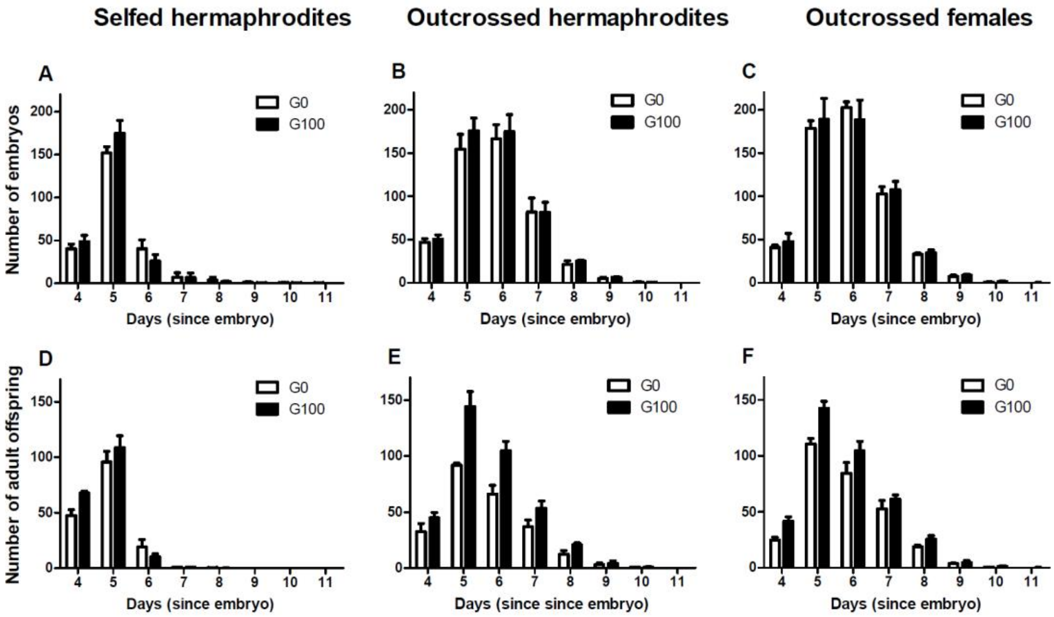

Supplementary Figure 1 - Longevity in the absence of mating

Bars represent the average individual fecundity (number of embryo progeny; top row) or fertility (number of adult offspring; bottom row) until death. Empty bars show results for samples of the ancestral populations (G0) and grey bars for derived populations after 100 generations of experimental evolution (G100). Individuals were enforced either to self or to outcross with tester males. Hermaphrodites came from male-hermaphrodite populations, which had maintained stable 50% of outcrossing during experimental evolution. Females came from male-female populations. Error bars denote one standard mean error among the three blocks done.

Supplementary Table 1. Sample sizes and summary statistics for each life-history trait measured in selfed hermaphrodites, outcrossed hermaphrodites and outcrossed females.

						day 4		day 5		day 6		day 7	
Treatment	Mating system	Generation	Population	Block	n	embryos	adult	embryos	adult	embryos	adult	embryos	adult
Selfed Hermaphrodites	Androdioecy	G0	A_0	b1	27	48.56 (24.83)	55.85 (29.68)	136.81 (37.70)	95.41 (37.93)	38.85 (32.31)	11.11 (12.51)	17.33 (23.78)	0.63 (1.78)
		G100	A_1	b1	29	58.45 (27.58)	67.21 (32.83)	143.79 (47.28)	96.00 (52.69)	42.07 (36.46)	6.90 (7.97)	18.38 (19.42)	0.41 (1.09)
		G0	A_0	b2	21	29.29 (18.28)	36.29 (22.01)	151.29 (51.66)	106.10 (44.72)	54.57 (38.62)	30.81 (24.17)	2.24 (4.94)	0.76 (1.64)
		G100	A_2	b2	16	34.50 (21.07)	60.81 (25.68)	186.94 (48.74)	128.62 (49.57)	25.38 (38.84)	17.50 (21.61)	2.19 (4.90)	1.50 (3.20)
		G0	A_0	b3	27	42.15 (13.07)	48.78 (13.17)	160.33 (53.89)	79.59 (27.47)	23.07 (21.99)	13.78 (13.84)	1.11 (2.34)	0.93 (1.73)
		G100	A_3	b3	26	51.00 (17.96)	70.88 (16.15)	189.96 (32.87)	110.92 (26.45)	15.27 (18.24)	10.15 (11.04)	0.50 (2.16)	0.38 (1.39)
Outcrossed Hermaphrodites	Androdioecy	G0	A_0	b1	23	57.74 (24.82)	45.13 (19.88)	133.26 (28.67)	97.61 (31.13)	143.91 (63.83)	81.09 (40.12)	69.57 (57.78)	32.87 (28.99)
		G100	A_1	b1	24	52.38 (26.79)	49.12 (24.60)	157.08 (39.37)	123.04 (32.49)	146.96 (69.76)	94.62 (46.40)	60.79 (51.41)	48.04 (35.65)
		G0	A_0	b2	28	39.86 (17.53)	19.71 (9.34)	148.14 (48.41)	90.14 (41.34)	182.46 (64.14)	75.14 (37.16)	119.82 (59.38)	53.75 (34.98)
		G100	A_2	b2	26	41.00 (18.28)	35.15 (15.24)	173.81 (62.00)	161.69 (61.31)	175.62 (67.09)	103.85 (51.81)	92.96 (72.95)	48.27 (40.57)
		G0	A_0	b3	25	48.36 (16.76)	32.20 (10.81)	189.64 (28.78)	92.56 (28.14)	195.56 (71.33)	51.76 (28.55)	70.16 (59.22)	32.44 (29.23)
		G100	A_3	b3	26	57.92 (22.56)	49.54 (16.62)	201.46 (36.62)	155.46 (32.55)	223.85 (61.17)	128.38 (48.37)	99.85 (58.82)	71.08 (41.55)
Outcrossed Females	Dioecy	G0	D_0	b1	22	35.00 (22.36)	29.36 (19.30)	164.14 (33.60)	115.14 (27.36)	203.00 (49.50)	100.27 (39.05)	116.14 (63.37)	66.55 (36.22)
		G100	D_1	b1	23	27.70 (19.72)	37.52 (24.98)	137.22 (49.54)	130.96 (41.88)	141.87 (59.08)	95.35 (49.85)	88.87 (71.09)	56.32 (45.57)
		G0	D_0	b2	27	42.56 (24.14)	22.22 (14.87)	171.26 (62.79)	111.22 (57.91)	186.70 (85.30)	65.26 (40.63)	103.44 (64.03)	38.96 (34.36)
		G100	D_2	b2	28	54.43 (24.34)	36.89 (16.30)	203.89 (42.97)	140.96 (55.60)	210.29 (80.86)	97.18 (59.71)	124.32 (82.44)	58.82 (47.13)
		G0	D_0	b3	25	42.84 (28.20)	21.48 (13.88)	194.88 (60.55)	100.48 (37.33)	213.88 (84.99)	85.84 (45.55)	87.72 (66.22)	50.64 (41.16)
		G100	D_3	b3	28	59.61 (29.24)	49.11 (23.59)	221.64 (75.79)	154.07 (57.64)	211.93 (103.48)	121.29 (67.25)	107.75 (72.54)	68.57 (49.07)

Notes: n indicates sample size after quality control of the data. Values show the mean per population sample with its standard deviation in parenthesis for daily embryo and adult progeny production, and lifespan.

Supplementary Table 1. (Cont.)

						day 8		day 9		day 10		day 11		
Treatment	Mating system	Generation	Population	Block	n	embryos	adult	embryos	adult	embryos	adult	embryos	adult	longevity
Selfed Hermaphrodites	Androdioecy	G0	A_0	b1	27	10.52 (14.87)	0.19 (0.79)	2.93 (6.57)	0.00 (0.00)	1.33 (5.00)	0.00 (0.00)	0.63 (3.27)	0.00 (0.00)	10.35 (4.42)
		G100	A_1	b1	29	4.10 (6.62)	0.10 (0.31)	1.31 (2.89)	0.00 (0.00)	1.00 (2.73)	0.00 (0.00)	0.00 (0.00)	0.00 (0.00)	12.19 (4.57)
		G0	A_0	b2	21	0.10 (0.44)	0.19 (0.68)	0.05 (0.22)	0.00 (0.00)	0.05 (0.22)	0.00 (0.00)	0.00 (0.00)	0.00 (0.00)	10.16 (3.24)
		G100	A_2	b2	16	1.38 (3.01)	0.25 (0.45)	0.06 (0.25)	0.06 (0.25)	0.00 (0.00)	0.00 (0.00)	0.00 (0.00)	0.00 (0.00)	11.86 (3.63)
		G0	A_0	b3	27	0.00 (0.00)	0.11 (0.32)	0.00 (0.00)	0.00 (0.00)	0.00 (0.00)	0.00 (0.00)	0.00 (0.00)	0.00 (0.00)	9.95 (2.89)
		G100	A_3	b3	26	0.00 (0.00)	0.00 (0.00)	0.04 (0.20)	0.04 (0.20)	0.00 (0.00)	0.00 (0.00)	0.00 (0.00)	0.00 (0.00)	15.30 (5.44)
Outcrossed Hermaphrodites	Androdioecy	G0	A_0	b1	23	18.52 (23.55)	10.87 (17.96)	3.83 (12.20)	0.09 (0.42)	1.09 (3.85)	0.26 (0.92)	0.09 (0.42)	0.17 (0.83)	7.48 (1.21)
		G100	A_1	b1	24	24.50 (31.04)	17.58 (23.80)	7.33 (17.14)	0.04 (0.20)	0.08 (0.41)	0.04 (0.20)	0.00 (0.00)	0.00 (0.00)	7.46 (1.35)
		G0	A_0	b2	28	30.93 (27.54)	20.21 (20.74)	8.75 (17.86)	5.36 (13.53)	1.21 (4.78)	1.04 (3.90)	0.04 (0.19)	0.04 (0.19)	8.00 (1.05)
		G100	A_2	b2	26	26.73 (27.55)	23.00 (23.03)	6.23 (12.58)	6.73 (14.02)	0.42 (2.16)	0.50 (2.55)	0.00 (0.00)	0.00 (0.00)	7.59 (0.96)
		G0	A_0	b3	25	19.52 (34.14)	9.32 (17.81)	4.04 (10.33)	3.24 (8.56)	0.60 (3.00)	0.36 (1.80)	0.00 (0.00)	0.00 (0.00)	7.20 (1.32)
		G100	A_3	b3	26	26.54 (30.67)	23.62 (24.44)	6.04 (10.30)	6.27 (10.90)	1.27 (4.23)	1.50 (5.02)	0.00 (0.00)	0.00 (0.00)	7.59 (1.50)
Outcrossed Females	Dioecy	G0	D_0	b1	22	35.73 (33.02)	18.59 (16.82)	9.55 (13.38)	2.91 (9.25)	0.18 (0.59)	0.00 (0.00)	0.00 (0.00)	0.00 (0.00)	7.67 (1.02)
		G100	D_1	b1	23	33.30 (34.16)	21.22 (22.72)	10.09 (22.50)	0.74 (2.56)	1.61 (5.21)	0.65 (2.55)	0.35 (1.47)	0.22 (0.85)	8.25 (1.37)
		G0	D_0	b2	27	31.78 (27.75)	20.78 (18.26)	7.52 (14.57)	4.74 (8.70)	1.33 (3.32)	0.89 (2.04)	0.00 (0.00)	0.00 (0.00)	7.77 (1.07)
		G100	D_2	b2	28	31.21 (34.66)	22.43 (25.97)	6.79 (11.28)	5.18 (9.20)	0.29 (1.01)	0.29 (1.33)	0.00 (0.00)	0.00 (0.00)	7.76 (1.23)
		G0	D_0	b3	25	31.20 (29.96)	16.68 (16.81)	4.60 (10.28)	3.16 (6.88)	0.44 (2.20)	0.32 (1.60)	0.00 (0.00)	0.00 (0.00)	7.15 (0.88)
		G100	D_3	b3	28	40.46 (34.03)	31.93 (25.82)	8.61 (17.68)	7.21 (13.60)	2.29 (9.03)	2.36 (9.39)	0.00 (0.00)	0.00 (0.00)	8.17 (1.23)
